# Life cycle assessment and cost-benefit analysis of nature-based solutions for contaminated land remediation: A mini-review

**DOI:** 10.1016/j.heliyon.2023.e20632

**Published:** 2023-10-05

**Authors:** Khaled Alshehri, Zhenghui Gao, Michael Harbottle, Devin Sapsford, Peter Cleall

**Affiliations:** a School of Engineering, Cardiff University, Cardiff CF24 3AA, UK; b Department of Civil Engineering, College of Engineering, University of Bisha, Bisha, 61922, P.O. Box 001, Kingdom of Saudi Arabia

**Keywords:** Nature-based solutions, Soil remediation, Groundwater remediation, Life cycle assessment, Cost-benefit analysis, Ecosystem services, Sustainability assessment

## Abstract

Nature-based solutions (NbS) have gained significant attention as a promising approach for remediating contaminated lands, offering multiple ecosystem services (ESs) benefits beyond pollution mitigation. However, the quantitative sustainability assessment of NbS remediation systems, particularly with regard to post-remediation impacts, remains limited. This mini-review aims to address the existing gaps in the assessment of NbS remediation systems by evaluating the limitations of life cycle assessment (LCA) and cost-benefit analysis (CBA) methodologies. A systematic literature search was conducted resulting in the review of 44 relevant studies published between 2006 and 2023. The review highlights an increasing trend in the coverage in the sustainability assessment literature of NbS remediation systems. Phytoextraction was identified as the main NbS mechanism employed in 65 % of the reviewed works, targeting contaminants such as heavy metals and hydrocarbons. However, the post-remediation aspects, including impacts on ESs and the end-of-life management of NbS biomass, were often neglected in the assessments with only a subset of studies partially exploring such aspects. The findings underscore the need for a comprehensive and integrated approach to assess the sustainability of NbS remediation systems, including the incorporation of economic factors, site-specific considerations, and post-remediation impacts. Addressing these gaps will enhance the understanding of NbS effectiveness and facilitate informed decision-making for contaminated land remediation.

## 1 Introduction

Land contamination could pose serious public health risks through exposure to contaminants released from soil [1]. It is also associated with decreased agricultural productivity and increased bioaccumulation of contaminants in crops rendering them unsafe for human consumption [2]. Remediation of contaminated land was found to have positive impacts on the health of children [3], increased access to public amenities [4], and increased economic growth [5]. Nature-based solutions (NbS) have emerged as a promising approach for contaminated land remediation (CLR), with the potential to provide multiple benefits beyond just mitigating the risk of exposure to pollutants [6]. NbS remediation systems are defined as "strategies inspired and supported by nature, simultaneously providing human well-being and biodiversity benefits” [7]. The impacts of remediation are classified as primary impacts pertaining to the impacts of contamination, secondary impacts related to remediation activities, and tertiary post-remediation impacts from reoccupying the land [8]. Life cycle assessment (LCA) and cost-benefit analysis (CBA) are two widely used methods for evaluating the environmental and economic performance of conventional remediation systems [9] as well as NbS systems [10]. Yet neither LCA nor CBA is capable of assessing the full extent of NbS remediation impacts [11,12]. Though the developments in NbS for contaminated land remediation have been comprehensively reviewed by ٍSong et al. [11] and more recently by Hou et al. [7], to our knowledge no other work has investigated the sustainability assessment literature of NBS for contaminated land remediation. This review elucidates the shortcomings of both LCA and CBA in the context of NbS remediation systems by exploring the following question: What are the existing gaps in the quantitative sustainability assessment of NbS remediation systems for contaminated land, with a specific focus on post-remediation impacts? The review is divided into 4 sections in addition to the introduction. Section 2. describes the search strategies, eligibility criteria, and synthesis of the results. Section 3 summarises the evidence across 4 sub-topics: section 3.1 reports on the NbS for CLR technologies used in the case studies; sections 3.2 and 3.3 explore the application of LCA and CBA to support decision-making of the NBS for CLR,respectively; section 3.4 examines the assessment of post-remediation impacts of NbS for CLR. Section 4 presents an overall discussion of the review's findings and limitations and discusses future research directions. Section 5 draws conclusions and recommendation from the reported evidence and results discussion.

## 2 Methods and materials

**Fig. 1:**
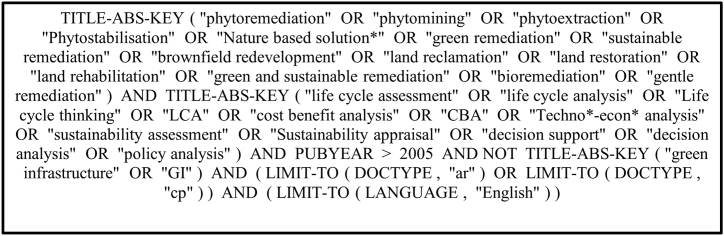
Search key terms.

**Fig. 2:**
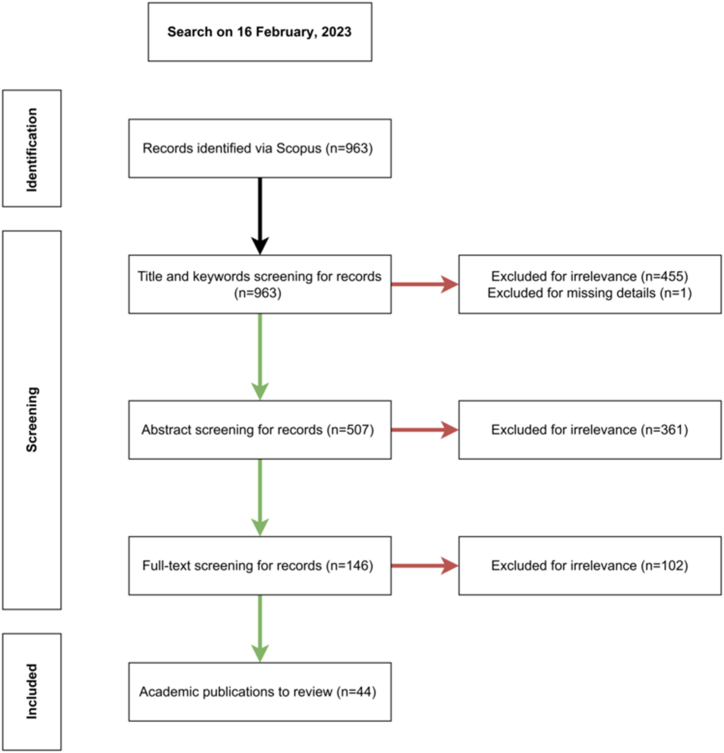
The screening process of the reviewed works.

A systematic literature search based on the PRISMA guidelines [13], was conducted on the Scopus database using key terms (see Fig. 1) to collect the recent studies on February 16, 2023 reflecting the inclusion and exclusion criteria listed in Table 1. The search was limited between January 2006 and February 2023 since the relevant LCA ISO standards 14040/14044 were released in 2006. To reduce the risk of bias, the search was limited to peer-reviewed documents and excluded grey literature sources (e.g., government reports, white papers and trade publications). The titles and authors' keywords of the initial lists (n = 963) were initially screened to exclude out-of-scope papers such as those relating to green infrastructure. An abstract screening step was undertaken to determine the relevance of the papers (n = 507) for a full-text screening (n = 146) prior to the data extraction phase (n = 44). The screening process and reviewed works are presented in Fig. 2 and Table B1 respectively. The data extraction is based on the data items (Table A.1 & A.2) designed to inform the study's research questions., the extracted data are then summarised and reported in spreadsheet format (see Table B2) to synthesise the review findings.

**Table 1:** Inclusion and exclusion criteria.

Criterion category	Criterion	Value
Inclusion	NbS application domain	Contaminated soil and groundwater remediation
	Sustainability assessment methodology	LCA or CBA
	Language	English
	Publication type	Peer-reviewed journal articles and conference papers
	Publication years	January 2006–February 2023
	Research database	Scopus
Exclusion	NbS application domain	Non-remedial application (e.g., green infrastructure for stormwater management)
	Publication type	Grey literature (e.g., trade publications)

## 3 Synthesis of the results

**Fig. 3:**
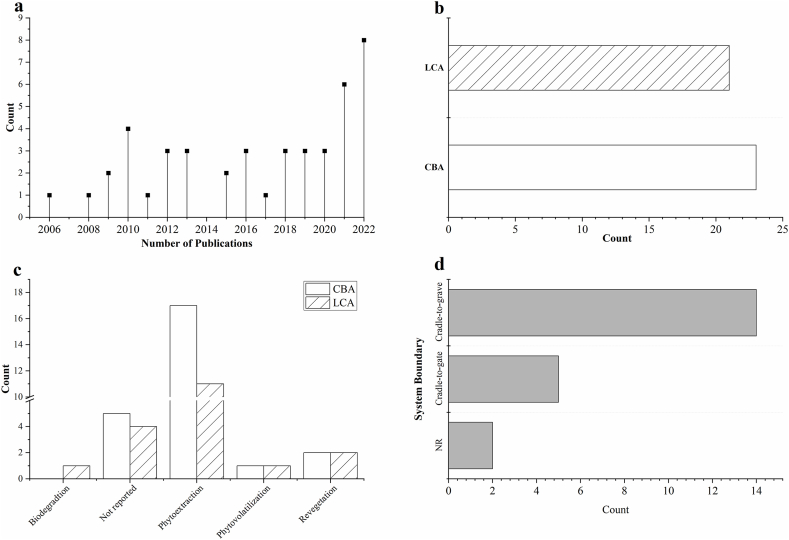
a) Total number of publications b) Number of works based on reported methodology c) Frequency of reported NbS mechanisms per applied methodology d) Frequency of defined system boundaries in reviewed LCAs.

A total of 44 studies were reviewed. As illustrated in Fig. 3a, there is a noticeable trend of expanding coverage in the literature, with the annual number of publications increasing to 8 in 2022. This represents a significant rise fromthe average of 2.6 publications per year prior to 2020*.* Twenty-three reviewed works (53.3 % of the reviewed works) utilised CBA while the remaining 47.7 % (n = 21) of the studies were LCA-based (see Fig. 3b).

### 3.1 Nature-based solutions (NbS) for contaminated land remediation (CLR)

NbS for contaminated land remediation is gaining traction recently [11]. NbS here encompasses both phytoremediation and passive bioremediation, active bioremediation systems such as biopiles were excluded from this work because such systems did not align with the NbS for CLR definition put forward by Hou et al. and quoted in section 1 (see section 4 for further discussion).

#### 3.1.1 Characteristics of NbS systems

Fig. 3c summarises the NbS mechanisms in the reviewed works including phytoextraction, phytovolatilization, phytostabilisation, and biodegradation by microorganisms. Effective remediation sometimes requires coupling several NbS mechanisms (e.g., phytoextraction and phytostabilisation) [14], phytoextraction and phytovolatilization [15] and/or applying intercropping species targeting different contaminants [16].

The nature of the contaminated materials largely determines which of the NbS mechanisms will achieve in use, soil decontamination in the reviewed works was achieved largely through phytoextraction while groundwater remediation is achieved through phytovotalisation (e.g., Refs. [17,18]). Major contaminants of concern are heavy metals such as Cd and Pb and hydrocarbons to a lesser extent [19]. The reported sources of contamination include historic industrial activities, illegal wastewater discharge and extended atmospheric deposition from nearby industrial activities. The NbS systems used several types of biota including grass such as Taiwanese chenopod and Napier grass [20], vetiver [21], trees species such as willow and poplar [[22], [23], [24]], Eucalyptus trees [18], and coastal she-oak [25]) and microorganisms (e.g. Pseudomonas mendocina) [26].

#### 3.1.2 Characteristics of NbS case studies

The remediation periods varied depending on the nature of the case study (simulated, lab-scale experiments or field trials), the scale of the experiment, the level of contamination, and the planned/intended land use of the site. Lab-scale experiments (<12 months) tend to be shorter than field experiments (averaged 6.7 years) since lab-scale experiments often focuses only on the NbS to uptake contaminants in controlled environments whereas field experiments are subject to variable environmental and economic factors [27]. Notably, the reclaimed mining area presented by Brunori et al. was the longest experimental field case study spanning 34 years whereas simulated cases varied between 2 and 32 years [28]. In-situ treatment was the prevailing remedial strategy while off-site treatment was reported in one study only which assesses the sustainability of reusing phytoremediated port sediment [29].

The site's area and location determine the feasibility of NbS remediation systems [14], therefore to ensure the economic feasibility of NbS remediation systems Suer et al. recommend the site area should be > 5000 m^2 17^ and ideally located in a marginal land (due to urban land competition) [22]. Geographically, case studies are concentrated in European countries and China. The reported European case studies are in historic industrial areas while agricultural land makes up most of the cases in China.

### 3.2 LCA

This section examines how LCA was applied to NbS for CLR sustainability assessment in the reviewed literature. LCA is used to inventory the energy and materials used and assess the environmental impacts over the life cycle of remediation systems [30]. LCA is employed in the remediation decision-making to model environmental impacts and/or optimize remediation systems for reducing potential impacts [31]. The first LCA application to a NbS remediation system was reported in 2009 by Suer et al. [17] to phytoremediate hydrocarbon-polluted sites by willow.

#### 3.2.1 Functional unit (FU) and system boundary

The LCA results are attributed to a functional unit (FU) which acts as a reference unit for the remediation system being considered and enables the comparison of multiple scenarios. The FUs of reviewed remediation LCAs are often defined as the remediation of a specific area or volume to a regulatory requirement [20,32] whereas in a few cases, the FU was defined as bioenergy potential [17,33], reflecting the goal of the particular LCA study. The scope of LCA analyses depends on the modeller's choice of system boundary namely: cradle-to-gate (66.7 % of the reviewed LCA studies), or cradle-to-grave (see Fig. 3d) and is also driven by the LCA objectives. A cradle-to-gate system boundary is selected when the focus is the bioenergy potential rather than the mitigation of contamination risk such as [29] whereas a cradle-to-grave system is defined in cases looking primarily at traditional remedial targets [34,35].

#### 3.2.2 LCI and LCIA

**Fig. 4:**
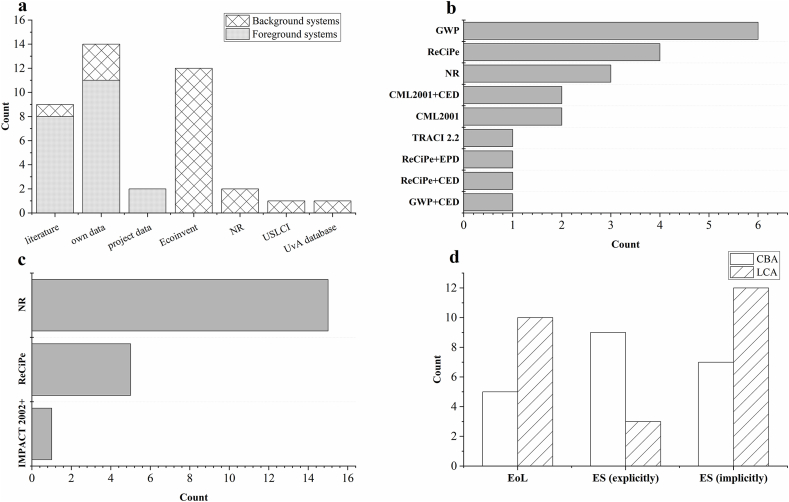
a) LCI of foreground and background systems b) Midpoint impact indicators c) Endpoint impact indicators d) Assessment of end-of-life management and ESs in the reviewed works.

The life cycle inventory (LCI) of remediation systems (foreground systems) was developed on primary data (own data or project data, n = 13), or literature (n = 8) amounting to 62 % and 38 % the reviewed LCAs respectively while the LCI of background systems were based on secondary sources predominantly from Ecoinvent (see Fig. 1a). Several life cycle impact assessment (LCIA) methods were employed such as ReCiPe, and CML2001. The most used LCIA mid-point indicators were the global warming potential (GWP) followed by the cumulative energy demand (CED),which measures the life cycle fossil fuel consumption, that is often assessed in tandem with mid-point indicators (see Fig. 4b). In earlier studies, the coupled application of CED and GWP reflected a major trend, focusing on carbon footprinting and leaving out other important LCIA indicators. Three case studies reported end-point LCIA indicators which were ReCiPe and IMPACT 2002+ (see Fig. 4c). SimaPro was the most reported LCA software (n = 9) followed by Sphera's GaBi (n = 5) making up 20.5 % and 11.4% of the reviewed LCAs, while older studies tended to be spreadsheet-based using primary data and/or limited literature information.

#### 3.2.3 Choice of reference scenario

The comparison of NbS systems to no-action and conventional remediation scenarios is a common theme in the reviewed works. The no-action scenario is often assumed as a monitoring system to monitor natural attenuation over long periods while the conventional remediation scenarios consisted of a dig-and-fill and/or other energy-intensive remedial alternatives (e.g., chemical stabilisation, and soil washing). The proximity to bioenergy plants increases the feasibility of NbS systems as the transport distance of biomass contributes significantly to GHG emissions. Economical elements were absent in most analyses except for the studies by Witters et al. [36], da S Trentin et al. [37], and O'Connor et al. [18]. Similarly, the site redevelopment/reuse planning was not considered in the reviewed works corroborating the results of previous review studies [31,38].

### 3.3 CBA

CBA of remediation aims to quantify the costs and benefits of remedial strategies to facilitate the remediation decision-making process. The remediation cost items include the capital expenditures (e.g., land acquisition, procurement of construction materials and ownership of remediation systems) and operational expenditures (e.g., personnel payroll, earthworks, transport activities, long-term site monitoring and supporting remediation activities) [10]. The remediation objectives (i.e., urban brownfield redevelopment and/or agricultural productivity improvement) define the beneficial items such as improved economic activity, and enhanced agricultural yield but could also include direct and indirect employment, reduced health risks and recreational opportunities [39]. The potential economic benefits of bioenergy provisioning were considered in most of the reviewed CBA works.

#### 3.3.1 Monetary unit of comparative analysis

In 74 % of the reviewed CBA studies (n = 23), a unit-price of remediation was assessed per m^2^ (soil) or m^3^ (soil/groundwater), ranging from $0.31 to $87.7 and averaging 19 $\frac{{USD}_{2022}}{m^{2}}$. In comparison, active bioremediation strategies unit prices ranged from $50.7 to $310.4 averaging 157.6 $\frac{{USD}_{2022}}{m^{2}}$ in Chile as reported by Orellana et al. [40], it is noteworthy that remediation costs are remedial objective and site specific; therefore direct comparison among literature unit-prices might lead to inaccurate conclusions [41]. In other cases, a benefit-cost (b/c) ratio was employed to compare different remedial scenarios without disclosing the monetary costs for example Demir et al. (2021) reported a b/c ratio for Pb removal ranging between 46.18 and 48.16 while ranging between 61.81 and 64.36 for Ni removal [42].

#### 3.3.2 Discounting rate

The choice of a discounting rate is an important aspect of CBAs reflecting the willingness of today's society to mitigate future risks at the expense of the wealth of the present (e.g., a discounting rate of 0 % indicates that cross-generational welfare is of equal importance from a present perspective) [43]. Only 11.4 % (n = 5) of the reviewed works reported a discounting rate, the reported discounting rates and examined duration were 3 % for 200 years [24], 5 % for 20 years [15], 5 % for 21 years [44], 9 % for 20 years [45], and 12 % for 3 years [25].

#### 3.3.3 Scope of CBAs

Few studies focused on the post-remediation economics such as the economic viability of contaminated willow biomass pyrolysis [45], potential farmer's additional income due to selling energy maize fodder to central anaerobic digestion facility while bearing the cost of fodder maize to feed onsite cattle [46], compared to the valorisation of a locally-owned plant [47]. While others focused exclusively on determining the appropriate land-use mix of planned brownfields remediation to maximize the ES value of non-food relates ESs [23].

### 3.4 Post-remediation impacts of NbS for CLR

As alluded to in sections 3.2.3, 3.3.3, the post-remediation impacts are sometimes overlooked in the LCA and CBA application of NbS remediation systems. Here we focus specifically on the end-of-life management of contaminated NbS biomass (section 3.4.1) and the consideration of ESs of NbS systems (section 3.4.2).

#### 3.4.1 End-of-life (EoL) management of NbS biomass

The end-of-life management of NbS biomass is a challenge that may undermine the advantages of NbS systems if not done properly [6]. For instance, the decomposition of NbS biomass in landfills could release contaminants of concern to the groundwater exacerbating the contamination risks [48]. Only 36.4 % (n = 16) of the 44 reviewed studies assessed aspects of EoL in their approach while one study discussed EoL qualitatively (see Fig. 4d). EoL was primarily considered in LCA analyses (27.2 %, n = 12) in the context of determining the most sustainable end-of-life management technology in terms of overall life-cycle impacts, the biomass transport distance to the treatment facility was found to be an important factor in the environmental feasibility of NbS biomass valorisation [49,50] as well as the used valorisation technology [51]. The examined EoL strategies were anaerobic digestion [20], composting [52], esterification [36], incineration [53] and landfilling [49].

CBA studies, as discussed above, focused on the economic feasibility of EoL strategies in terms of improved agricultural outputs and/or bioenergy yield potential. For instance, Gou et al. examined the economic benefits of intercropping phytoaccumalator species (*Hylotelephium spectabile* & *Amaranthus hypochondriacus*) with cash crops (rapeseed/maize) rotation while adhering to national regulations of acceptable heavy metals concentration in the biomass of cash crops, it was found that the total economic profits were up to 123.5 % higher than wheat crops [16], because wheat's uptake of heavy metals is higher making wheat unsafe for human consumption and economically unviable. Meanwhile, the assessments of bioenergy potential explored the economic profits of pyrolysis of willow biomass to produce fuel oil to be used in a combined heat and power plant [45]. Another example is energy maize production for anaerobic digestion under two scenarios; In the first scenario: the farmer will sell energy fodder grown onsite and continue raising cattle fed clean fodder procured offsite [46], and in the second scenario adds the potential income of managing a local digester cooperative, i.e. among a group of farmers [47]. It is noteworthy that the economic viability of NbS valorisation is driven by the potential income of the intended end use (e.g., feedstock of energy valorisation), the regulatory standards of contaminants concentrations and the size of remediated areas (determining the potential yield).

#### 3.4.2 Accounting of ESs

ESs refer to the benefits that people derive from ecosystems [54]. These benefits include provisioning services like biomass production, regulating and control services such as carbon sequestration, and cultural services like recreational activities [55]. NbS remediation systems provide several ESs such as provisioning bioenergy, biotic degradation of contaminants, and aesthetic sceneries. That said, the impacts on ESs are tertiary impacts of remediation (i.e. takes place post the remedial action) and are rarely considered explicitly within LCAs of soil remediation [11,38]. In this review, we explored the patterns of accounting of ESs whether explicitly (i.e., clearly defined as ESs) or implicitly (assessed in terms of economic terms rather than the impacts on local ecosystems). We found out that only 27.7 % (n = 12) of the 44 reviewed studies have explicitly assessed the impacts of NbS systems on local ESs, 45.5 % (n = 20) of the studies considered ESs implicitly while the remaining 27.7 % (n = 12) of the studies did not consider any aspect of ES (see Fig. 4d).

Three LCAs investigated the NbS remediation impacts on ES amounting to 6.8 % of the reviewed studies. Brunori et al. considered the carbon balance of an oak plantation over a reclaimed mining site by carbon footprinting the plantation management activities compared to the carbon stocks in oak biomass over the period between Nov. 1979 and Feb. 2014 ^28^, while O'Connor et al. modelled the carbon stored in eucalyptus tree planted in a phytoremediated contaminated groundwater table [18]. Chen et al. developed a novel mid-point LCIA indicator that characterises the impacts on the soil organic content (SOC) due to land use change, SOC is a proxy for the soil functionality to provide some ESs such as net primary production, and improved biodiversity [26], but it fails to consider several other ESs such air filtration and cultural ESs. Notably, the LCA studies focused solely on carbon storage and sequestration leaving out other important ESs.

Evidently, the nine CBAs (20.5 % of reviewed works) assessing ESs focused on the effects of NbS remediation on the economic benefits of ESs mostly bioenergy provisioning, food provision, and carbon sequestration. However [56], proposed a CBA-based multicriteria decision analysis (MCDA) remediation sustainability framework incorporating several ESs indicators, which was applied semi-quantitatively on a broad range of remedial technologies including bioremediation alternatives based on literature. An expanded framework which assessed the uncertainty of ES valuations was applied to a 40,000 m^2^ site contaminated by polycyclic aromatic hydrocarbons (PAH) and heavy metals including zinc, copper and lead [24]. Though the study succeeded in assessing impacts on ESs comprehensively, it overlooked other important parameters such as the LCIA impacts of NbS remediation. The remaining seven studies are classical CBA applications of potential economic benefits such as comparative economic feasibility to degrade contaminants [21,57], bioenergy potential [58], improved agricultural production [23,59,60], and carbon sequestration and storage [61,62].

## 4 Overall discussion, limitations, and future outlook

The main objective of this review is to highlight gaps in the quantitative sustainability assessment of NbS remediation systems of contaminated land and in particular the post-remediation impacts. LCA and CBA have been commonly used in remediation planning and decision-making [9], therefore LCA and CBA were selected as the methodologies of focus in this review. The reported NbS systems depended mostly on phytoremediation mechanisms while only one case study utilised microorganisms for remediation. This could be attributed to the selection of keywords in the literature search, though bioremediation systems (bacteria and fungi-based) could be considered as an NbS system however such systems include the use of machinery during operations such as aeration fans, and/or earthworks to manage the land incurring additional costs [18]. The minimal maintenance care of phytoremediation systems is a substantial advantage while offering additional benefits, and thus more closely aligns with the definition of NbS, hence is selected as the focus of this review.

The feasibility of NbS systems is determined by the type and concentration of contaminants, proximity of receptors, regulatory standards, area and location of the site, and the planned use of the site. The choice of suitable NbS species is driven by the type of contaminants, but preference should to native species to avoid disturbing the local biodiversity balance. The efficacy of remediation is an important factor as NbS systems tend to be slower than their conventional counterparts hence NbS systems might be better suited to marginal lands.

LCA and CBA were applied equally in the reviewed works with varying assessment scopes. The EoL management of biomass was assessed mostly in LCAs whereas the studies that considered ES were CBA-based. Though the LCA by O'Connor and colleagues did compare the cost and net environmental benefits of NbS systems relative to enhanced bioremediation it only considered carbon storage in the form of timber production [18]. The CBA-based assessment by Volchko et al. is by far the most comprehensive attempt to consider the impacts of NbS remediation on ES and EoL management of NbS biomass [24], however, it does not account for the life cycle impacts of the NbS systems leaving out some potential environmental externalities.

As discussed above and in section 3.4, the reported LCAs and CBAs focused on different aspects of the post-remediation impacts reflecting the objectives of the sustainability assessment. Combining elements of CBA and LCA to account for the impacts on ES and life cycle impacts of EoL is necessary to improve our understanding of the performance of NbS systems. An integrated LCA-CBA approach could provide a more holistic approach to the sustainability of NbS for CLR facilitating the remediation decision-making process. It is noteworthy that other MCDA approaches have been used to assess the sustainability of NbS systems, but they often rely on qualitative and semi-quantitative measures which fall outside the quantitative scope of this review.

The main limitation of this review is inadvertently overlooking some of the emerging contaminants and recent technological developments of NbS for CLR, the reason being that such advancements were not yet reported in the reviewed literature which focused on the sustainability assessment practice rather than the state-of-the-art of NbS for CLR. A second limitation of this review is that theoretical framework proposals (i.e., not applied to case studies) were not investigated as missing comparative results does not align the quantitative scope of this review. Another point to consider is that there have been recent developments in emerging contaminants and novel NbS for CLR as Hou et al. [7]. However, these developments were not assessed in this review because they have not yet been considered in the sustainability assessment literature.

Based on the results of this review, we suggest focusing on the following areas for future research: 1) accounting of ESs beyond biomass (edible and non-edible) provisioning in the NbS for CLR sustainability assessment, 2) integration of LCA and CBA into comprehensive assessment frameworks, 3) assessment of emerging contaminants and novel NbS for CLR systems. This review has identified the gaps in knowledge and practice of sustainability assessment of NbS for CLR. These outcomes should guide forthcoming efforts in decision-making support for NbS in CLR.

## 5 Conclusion

We systematically reviewed the NbS for CLR sustainability assessment literature to identify current knowledge gaps and explore potential research avenues. Through a concise synthesis of the findings, we shed light on the technical aspects of the reported NbS systems and case studies, including quantitative sustainability assessment through LCA and/or CBA, and the post-remediation impacts on ESs and EoL management of NbS biomass.

In section 3.1, we examined the reviewed NbS systems in terms of flora/fauna species, NbS remediation mechanisms, and characteristics of the NbS reported case studies. We dedicated sections 3.1, 3.3 to the application of LCA and CBA methodologies to assess the sustainability of NbS remediation systems, highlighting the advantages and shortcomings of each methodology. In section 3.4, we focused on the sustainability assessment of two aspects of the post-remediation impacts of NbS systems, namely, the EoL management of NbS biomass and accounting of ES. Section 4 presents an overall discussion of the review findings and limitations and proposes key research questions for future efforts.

The increasing application of NbS systems to remediate contaminated lands should be coupled with the development of a comprehensive sustainability assessment framework to facilitate the NbS remediation decision-making process. Hence the motivation to conduct this concise overview of the quantitative sustainability of NbS for CLR.

## Funding

This work was carried out without receiving any dedicated grant or funding.

## Data availability statement

Data included in article/supplementary material/referenced in article.

## CRediT authorship contribution statement

**Khaled Alshehri:** Conceptualization, Data curation, Formal analysis, Methodology, Visualization, Writing – original draftWriting – original draft. **Zhenghui Gao:** Data curation, Formal analysis, Writing – review & editingWriting – review & editing. **Michael Harbottle:** Conceptualization, Methodology, Supervision, Writing – review & editingWriting – review & editing. **Devin Sapsford:** Conceptualization, Methodology, Supervision, Writing – review & editingWriting – review & editing. **Peter Cleall:** Conceptualization, Methodology, Writing – review & editingWriting – review & editing.

## Declaration of competing interest

The authors declare that they have no known competing financial interests or personal relationships that could have appeared to influence the work reported in this paper.

## Abbreviations

CBA: Cost-benefit analysis
CED: Cumulative energy demand
CLR: Contaminated land remediation
EoL: End-of-Life
ES: Ecosystem service
FU: Functional unit
GHG: Greenhouse gas
LCA: Life cycle assessment
LCI: Life cycle inventory
LCIA: Life cycle impact assessment
MCDA: Multi-criteria decision analysis
NbS: Nature-based solutions
PRISMA: the Preferred Reporting Items for Systematic Reviews and Meta-Analyses
SRC: Short-rotation coppice
SOC: Soil organic content
