# Supplementary material for: Life cycle assessment and cost-benefit analysis of nature-based solutions for contaminated land remediation: A mini-review
Source: Heliyon. 2023 Oct 5;9(10):e20632. doi: 10.1016/j.heliyon.2023.e20632 (PMC10569992; doi:10.1016/j.heliyon.2023.e20632)
Supplement: Multimedia component 1 [file mmc1.docx]

Supplementary Material

Life Cycle Assessment and Cost-benefit Analysis of Nature-based Solutions for Contaminated Land Remediation: a mini-review

**Khaled Alshehri^1,2*^, Zhenghui Gao^1^, Michael Harbottle^1^, Devin Sapsford^1^, Peter Cleall^1*^**

^1^School of Engineering, Cardiff University, Cardiff CF24 3AA, UK

^2^Department of Civil Engineering, College of Engineering, University of Bisha, Bisha, 61922, P.O. Box 001, Kingdom of Saudi Arabia

**Correspondence:** Khaled Alshehri: AlshehriKM@cardiff.ac.uk

# Supplementary Figures and Tables

## Supplementary Tables

Table A. 1 Data items charting of the bibliographic information, case study details and NbS remediation system

| Bibliographic information | Author(s) |
| --- | --- |
|  | Year of publication |
|  | Title of publication |
|  | Type of publication |
|  | Journal/ conference title |
| Case study details | Location |
|  | Latitude |
|  | Longitude |
|  | Type of study: lab-scale, field, or simulated |
|  | Area |
|  | Historic land use |
|  | Contaminants of concern |
| NbS remediation system | NbS species |
|  | NbS mechanism(s) |
|  | Remediation period |
|  | Remedial target |

Table A. 2 Data items charting for LCA, CBA, and post-remediation impacts

| Life cycle assessment(LCA) | Functional unit |
| --- | --- |
|  | System boundary |
|  | LCI source of foreground systems |
|  | LCI source of background systems |
|  | Life cycle impact assessment (LCIA) method |
|  | LCIA indicators |
|  | LCA software |
| Cost-benefit analysis(CBA) | Cost items |
|  | Discounting rate |
|  | Unit cost per m^2^ |
|  | currency |
|  | Fiscal year |
|  | Unit cost (USD_2022_ equivalent) |
| Post-remediation impacts | Discussion of End-of-life (EoL) management |
|  | EoL strategies |
|  | Discussion of ecosystems services (ESs) |
|  | Reported ESs |
